# Clinical evidence of acupuncture for infertile women with diminished ovarian reserve undergoing IVF/ICSI: a systematic review and meta-analysis

**DOI:** 10.3389/fendo.2026.1840157

**Published:** 2026-06-10

**Authors:** Liangliang Xu, Xueyan Liang, Wei Wang, Ruifang Zhang, Guangyao Lin, Kaili Cao, Ping Sun, Li Fan

**Affiliations:** 1 Department of Gynecology and Obstetrics, Affiliated Hospital of Gansu University of Chinese Medicine, Gansu, China; 2 National Clinical Research Center for Obstetrics and Gynecology (Peking University Third Hospital), Beijing, China

**Keywords:** acupuncture, diminished ovarian reserve, ICSI, infertility, IVF

## Abstract

**Background:**

Diminished ovarian reserve (DOR) is a major cause of infertility and remains a core challenge in reproductive medicine. Although previous studies have suggested that acupuncture may improve reproductive outcomes in infertile women with DOR undergoing *in vitro* fertilization (IVF) or intracytoplasmic sperm injection (ICSI), findings remain inconsistent. This meta-analysis therefore aimed to synthesize evidence from randomized controlled trials (RCTs) to evaluate the efficacy of acupuncture in these women undergoing IVF/ICSI.

**Methods:**

Eight databases were systematically searched for eligible RCTs up to February 28, 2026. The findings were presented in forest plots, with one primary outcome and ten secondary outcomes. Sensitivity analyses were carried out to assess the robustness of the pooled estimates. Subgroup analyses were conducted to explore whether the efficacy of acupuncture on the primary outcome was associated with the type of acupuncture or the number of acupoints adopted per treatment. Evidence grading was performed using the GRADEpro GDT.

**Results:**

11 RCTs involving 885 infertile women with DOR undergoing IVF/ICSI were finally included in the meta-analysis. The pooled evidence showed that acupuncture remarkably increased clinical pregnancy rate by 25% (risk difference [RD] = 0.25, 95% CI: 0.18 to 0.32; *p* < 0.00001), live birth rate by 22% (RD = 0.22, 95% CI: 0.05 to 0.40; *p* = 0.01), and embryo implantation rate by 30% (RD = 0.30, 95% CI: 0.20 to 0.41; *p* < 0.00001). In addition, acupuncture effectively improved the number of oocytes retrieved (MD = 1.72, 95% CI: 1.03 to 2.41; *p* < 0.00001), the number of optimal embryos (MD = 1.50, 95% CI: 1.42 to 1.58; *p* < 0.00001), and E_2_ on the day of hCG trigger (MD = 318.48, 95% CI: 137.16 to 499.80; *p* = 0.0006). Subgroup analysis further substantiated the value of acupuncture in improving clinical pregnancy rate. Sensitivity analyses confirmed the robustness of the pooled estimates. Evidence certainty for all assessed outcomes ranged from very low to moderate.

**Conclusions:**

Based on this pooled clinical evidence, acupuncture may be a beneficial intervention for improving clinical outcomes in women with DOR undergoing IVF/ICSI, although confirmation by future rigorously designed RCTs is warranted.

**Systematic Review Registration:**

https://www.crd.york.ac.uk, identifier CRD420261330054.

## 1 Introduction

Infertility constitutes a prominent global public health challenge, with the reported prevalence ranging from 3.5% to 16.7% in more developed countries and from 6.9% to 9.3% in less developed regions, corresponding to an overall median prevalence of 9% (1). The global burden of infertility has risen markedly, and it is estimated that this condition affects approximately 186 million individuals worldwide in the 21st century (2). Geographically, the prevalence of infertility remains notably high across multiple regions: for example, the prevalence reaches 29.6% in Côte d’Ivoire, 25% in China, and 24.5% in Kenya (3, 4). In response to the escalating public health burden of infertility, an increasing number of countries are taking urgent action to implement targeted infertility management policies, including China, Japan, the United Kingdom, and the United States (5, 6). These initiatives reflect an emerging global policy tendency to integrate fertility treatment into the framework of universal health coverage.

The etiology of infertility remains largely elusive, encompassing ovulatory dysfunction, endometriosis, uterine abnormalities, and other multifactorial etiologies (7). Diminished ovarian reserve (DOR) constitutes a primary etiological factor for infertility (7). Notably, 91% of women with DOR experienced infertility-related health impacts (8). Recent data from more than 180,000 ART cycles indicate that the overall prevalence of DOR ranges from 19% to 26% (9). Moreover, women with DOR are significantly associated with unfavorable reproductive outcomes, including an increased risk of miscarriage (10), and elevated risk of cycle cancellation in *in vitro* fertilization (IVF) (11). Despite the application of various adjuvant agents and controlled ovarian stimulation protocols to improve reproductive outcomes in infertile women with DOR, achieving effective therapy remains a substantial clinical challenge (12–14). For example, a recent meta-analysis involving 4,182 women with DOR confirmed that the novel PPOS protocol can effectively improve IVF/ICSI outcomes, including a reduction in the premature LH surge rate and cycle cancellation rate, as well as an increase in optimal embryos rate, cumulative pregnancy rate, clinical pregnancy rate; still, this protocol fails to improve live birth rate, embryo implantation rate, number of oocytes retrieved, and early miscarriage rate (12). Additionally, pretreatment with coenzyme Q10 represents another promising strategy for improving the number of retrieved oocytes and the clinical pregnancy rate, yet it is unknown whether coenzyme Q10 is effective in enhancing the embryo implantation rate (13). Thus, it is imperative for fertility clinicians to explore optimized therapeutic strategies for improving IVF/ICSI outcomes in these unique populations.

In Australia, 88.1% of women with DOR were advised to use complementary integrative medicine, including acupuncture, to enhance fertility outcomes (8). Acupuncture, a promising complementary strategy, has been widely applied in the clinical management of reproductive disorders, with accumulating evidence suggesting its therapeutic benefits (15, 16). A recent systematic review pooled the therapeutic effects of acupuncture in women with DOR and confirmed that acupuncture improves hormone levels, antral follicle count, ovarian blood supply and pregnancy outcomes, and these beneficial effects are associated with regulation of key signaling pathways including PI3K/AKT/mTOR and Nrf2/HO-1, inhibition of inflammatory responses, and reduction of apoptosis (17). Besides, acupuncture therapy has also been shown to alleviate anxiety and depression, which are common in women with DOR, thereby improving mental well-being alongside ovarian function (18). Overall, acupuncture serves as a safe, low cost, non-pharmacological intervention that enhances psychological health, ovarian function, and IVF outcomes. Importantly, leading organizations, such as the Chinese Reproductive Medicine Group, suggest acupuncture to enhance ART outcomes. Consequently, emerging randomized controlled trials (RCTs) have recently been carried out to evaluate the value of acupuncture in infertile women with DOR undergoing IVF/ICSI (19). Nevertheless, the findings remain conflicting across several studies. For example, Ma et al. (20) found that acupuncture dramatically improved the number of optimal embryos and oocytes retrieved, a finding contrary to that of Gou et al. (21). In addition, Zheng et al. (22) observed that acupuncture effectively reduced the duration and total dose of Gn used during ovarian stimulation. Conversely, another study (23) reported that acupuncture did not significantly reduce these parameters. These conflicting findings may stem from the limited sample sizes and single-center designs of individual studies. Therefore, this meta-analysis was carried out to systematically synthesize available RCTs and provide high-quality evidence for clinical practice. The objective of this meta-analysis was to assess whether acupuncture improves IVF/ICSI outcomes in infertile women with DOR.

## 2 Materials and methods

This study (PROSPERO registration No. CRD420261330054) was conducted in accordance with the PRISMA guidelines (24).

### 2.1 Search strategy

Two researchers (the first two authors) performed a systematic literature search in eight electronic databases from inception to February 28, 2026. The databases included four Chinese databases (Wanfang, SinoMed, VIP Information, and CNKI) and four English databases (PubMed, Embase, Cochrane Library, and Web of Science). Combinations of the following search terms were used: “diminished ovarian reserve”, “decreased ovarian reserve”, “declined ovarian reserve”, and “assisted reproduction technology”, “ICSI”, “IVF”, “electro-acupuncture”, “acupuncture”, “manual acupuncture”, and “transcutaneous electrical acupoint stimulation (TEAS)”. No restrictions were imposed on publication status or country of origin. Two reviewers independently screened the retrieved records for eligibility by assessing abstracts and full texts. Reference lists of included studies were further hand-searched to identify additional eligible literature. Any discrepancies between the reviewers were resolved through discussion or by consulting the corresponding author if necessary.

### 2.2 Inclusion and exclusion criteria

Inclusion criteria: (I) the study design was a RCT; (II) the participants diagnosed with DOR according to the well-recognized diagnostic criteria (25), defined as FSH ≥ 10 IU/L or AFC < 5~7 or AMH < 1.1 ng/mL; (III) all participants underwent IVF or ICSI, regardless of the ovarian stimulation protocol; (IV) The interventions were acupuncture or TEAS, with no restrictions on treatment frequency or duration. As a form derived from traditional acupuncture, TEAS was regarded as an acupuncture analogue intervention that shares the same peripheral neuromodulation mechanism (19); and (V) studies were published in English or Chinese. Articles in which acupuncture was administered as an adjunct to IVF/ICSI cycles were eligible, provided that the control group received identical concomitant treatments.

Exclusion criteria: (I) participants with reproductive system tumors, chromosomal abnormalities, or intrauterine adhesions; (II) research articles were self-controlled or cohort studies; (III) conference, reviews, animal studies, duplicate publications, protocols, or case reports; (IV) studies with unavailable full text, and (V) acupoint catgut embedding was excluded because it delivers continuous acupoint stimulation over several days, which differs substantially from the session-based stimulation of acupuncture or TEAS and may introduce clinical heterogeneity.

### 2.3 Data extraction and risk of bias assessment

Data extraction was processed independently by two reviewers and subsequently cross-checked to ensure accuracy. The extracted information was as follows: study population characteristics (including sample size, age, body mass index, and duration of infertility), details of interventions, and IVF/ICSI outcomes. The primary outcomes of interest were clinical pregnancy rate. Secondary outcomes included live birth rate, number of oocytes retrieved, embryo implantation rate, number of optimal embryos, duration of Gn used, total dose of Gn used, E_2_ on the day of hCG, resistance index, pulsatility index, and systolic/diastolic ratio of the ovarian arteries. Furthermore, the quality of enrolled articles was assessed using the Cochrane Collaboration tool. Each RCT was classified as having low, unclear, or high risk of bias. Disagreements were adjudicated by the corresponding author when required.

### 2.4 Statistical analysis

Review Manager 5.3 was used for all statistical analyses. The pooled estimates were presented using forest plots. The risk difference (RD) with 95% confidence intervals (CIs) was calculated for dichotomous outcomes, and mean difference (MD) with 95% CIs for continuous outcomes. According to the *Cochrane Handbook* (www.cochrane.org/authors/handbooks-and-manuals/handbook), RD provides more directly relevant clinical information than relative measures and is particularly useful for weighing benefits against harms. Although RD can be sensitive to baseline risk variation, the primary aim of this analysis was to estimate the absolute treatment effect. Heterogeneity was computed using the I^2^ statistic. A fixed-effects model was adopted if low heterogeneity was observed (I^2^ < 50%); while a random-effects model was utilized otherwise. A subgroup analysis was performed to explore whether the effect of acupuncture on the primary outcome was associated with the type of acupuncture or the number of acupoints used per treatment. Sensitivity analyses were carried out by sequentially omitting individual articles to test the robustness of pooled results, and were only applied when the meta-analysis included > 3 studies. For all statistical tests, a two-sided p value < 0.05 was deemed statistically significant.

### 2.5 Overall quality of the evidence

The certainty of clinical evidence for each pooled outcome was evaluated according to the GRADE criteria. Evidence certainty was classified into four hierarchical levels: high, moderate, low, and very low. The summary of findings table was generated through the GRADEpro GDT platform (https://gdt.gradepro.org).

## 3 Results

### 3.1 Included studies

**Figure 1:**
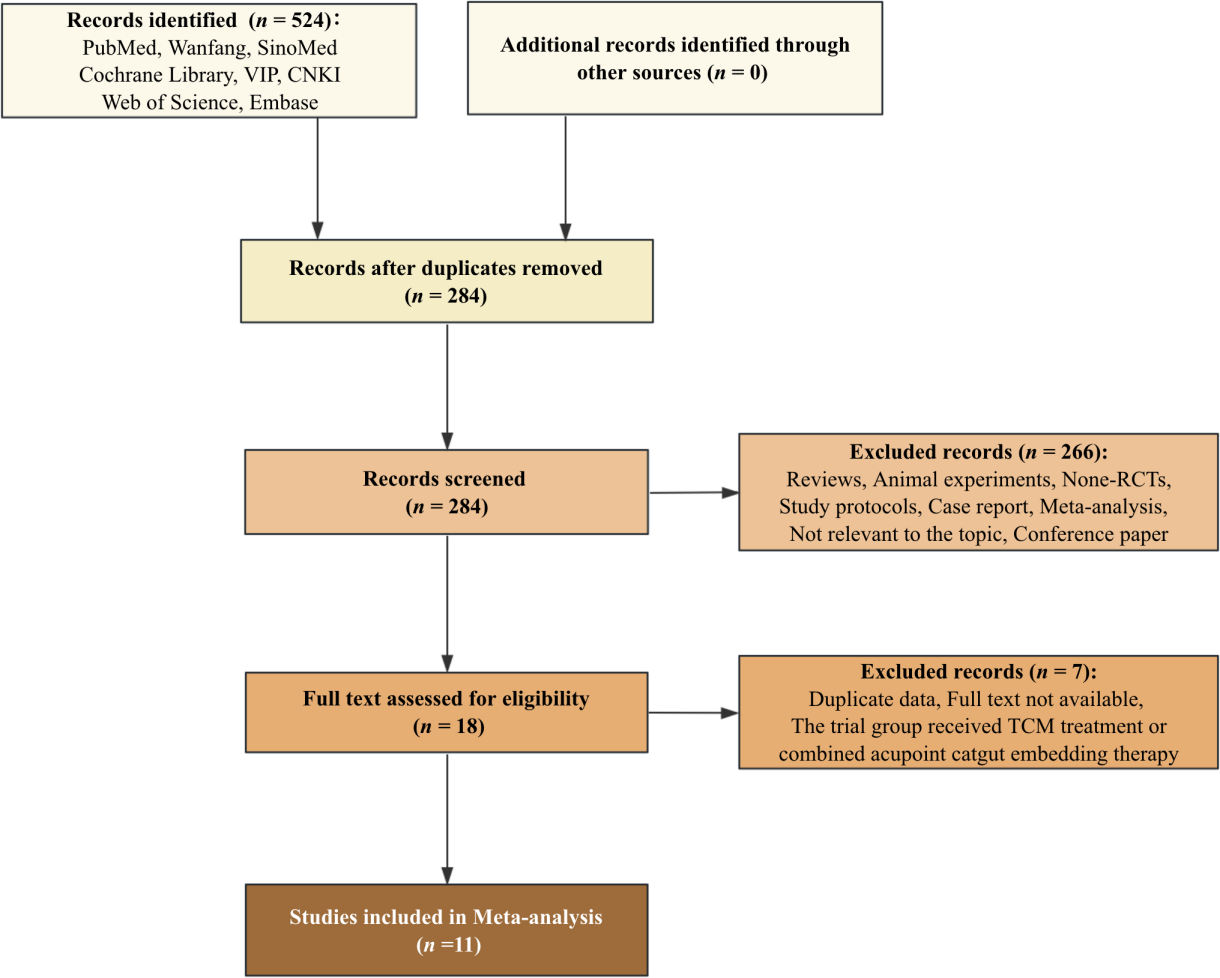
Flow diagram.

The process of study selection is presented in **Figure 1**. A total of 524 records were identified from eight databases. After removing 240 duplicates, 284 records remained. Following title and abstract screening, 266 records were excluded, leaving 18 full-text articles to be assessed for eligibility. Subsequently, following full-text review of 18 articles, 7 were excluded due to unavailability of the full text, duplicate data, or the control group received Chinese herb treatment or combined acupoint catgut embedding therapy. Finally, 11 RCTs were included in the quantitative analysis.

### 3.2 Study characteristics

This meta-analysis included 11 RCTs involving 885 women with DOR who underwent IVF or ICSI in China. Across the included RCTs, a total of 439 participants were assigned to the acupuncture groups and 446 to the control groups. The publication years of the included RCTs ranged from 2015 to 2025. Regarding acupuncture interventions, seven RCTs administered manual acupuncture to women in the trial groups, two used TEAS, and two RCTs applied either electroacupuncture or manual acupuncture combined with TCM before IVF/ICSI, respectively. For control conditions, seven studies employed a waitlist control, two administered placebo TEAS, and two implemented lifestyle counseling and TCM, respectively. In terms of ovarian stimulation protocols, five studies applied a minimal stimulation protocol, three used hormone replacement therapy, two adopted an antagonist protocol, and one did not specify the protocol used. The number of acupoints applied ranged from 5 to 21, and the treatment duration varied from two weeks to three menstrual cycles (**Supplementary Table 1**). Additional trial characteristics, including participant age, body mass index, and duration of infertility, are summarized in **Table 1**.

**Table 1:** Study characteristics.

Study	Year	Sample size (*n*)		Age (years)		BMI (Kg/m^2^)		Duration of infertility (years)		Outcomes
		Control	Trial	Control	Trial	Control	Trial	Control	Trial	
Ma (A) (20)	2025	30	30	38.40 ± 3.36	37.20 ± 3.97	NR	NR	2.22 ± 1.11	2.41 ± 0.90	①③⑤
Ju (26)	2024	33	33	36.97 ± 3.41	35.41 ± 3.54	22.66 ± 1.85	22.42 ± 2.26	4 (2, 5)	3 (1.5, 5.5)	①②③⑤⑥⑦⑧
Zhao (27)	2024	36	37	33.06 ± 3.40	33.19 ± 3.67	21.48 (20.66, 24.34)	21.48 (19.37, 22.97)	3.00 (1.00, 6.00)	3.00 (2.00, 7.00)	③⑧
Qin (23)	2024	38	37	36.5 ± 7.5	35.5 ± 6.8	22.53 ± 3.75	22.31 ± 3.21	4.44 ± 2.89	5.65 ± 2.21	①③④⑤⑨⑩⑪
Wen (A) (28)	2023	50	50	33.66 ± 3.86	33.84 ± 3.69	23.81 ± 2.98	24.97 ± 4.13	3.5 (2, 6)	3.0 (2, 7)	①②⑧⑨⑩⑪
Wen (B) (29)	2023	60	60	32.92 ± 5.13	32.90 ± 4.90	NR	NR	5.13 ± 3.69	4.47 ± 3.73	③
Ma (B) (30)	2023	50	50	35.49 ± 1.78	36.23 ± 1.26	21.38 ± 1.69	22.12 ± 1.18	3.13 ± 0.97	3.22 ± 1.21	①
Shen (31)	2022	33	32	37 ± 7	35 ± 6	22.7 ± 3.4	22.9 ± 3.8	4.5 ± 4.4	5.9 ± 4.3	①②③④⑥⑦
Gou (21)	2019	27	24	34.22 ± 4.64	34.46 ± 5.32	21.99 ± 2.81	22.75 ± 2.71	3.59 ± 1.58	3.62 ± 1.47	①③⑤⑥⑦
Zhou (32)	2016	33	30	36 ± 5	35 ± 5	NR	NR	6.9 ± 5.2	5.3 ± 4.6	①④
Zheng (22)	2015	56	56	36.88 ± 4.65	36.05 ± 5.48	23.56 ± 2.63	24.14 ± 4.32	4.75 ± 2.64	4.44 ± 2.98	①③④⑥⑦⑨⑩⑪

NR, Not reported; ① Clinical pregnancy rate; ② Live birth rate; ③ Number of oocytes retrieved; ④ Embryo implantation rate; ⑤ Number of optimal embryos; ⑥ Duration of gonadotropin used; ⑦ Total dose of gonadotropin used; ⑧ E_2_ on the day of hCG; ⑨ Resistance index; ⑩ Pulsatility index; ⑪ Systolic/Diastolic ratio.

### 3.3 Risk of bias assessment

**Figure 2:**
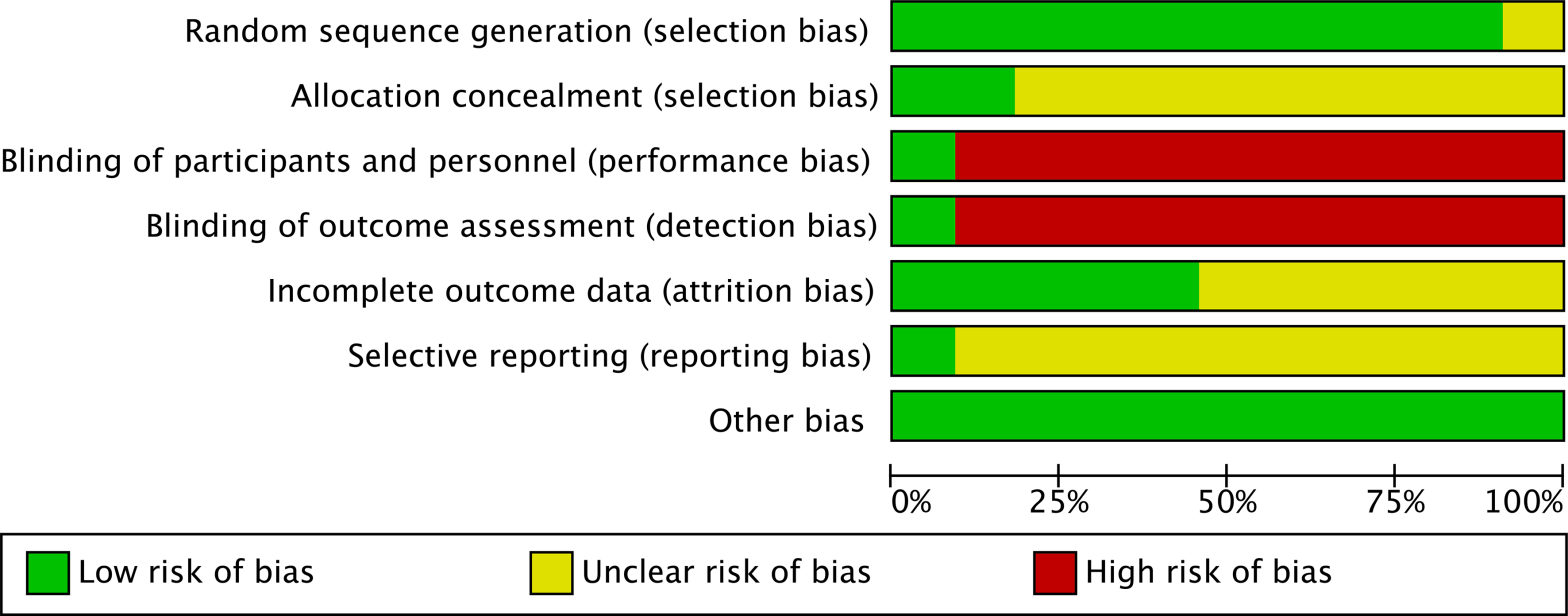
Risk of bias summary.

All included RCTs reported the use of randomization. Regarding sequence generation, most studies provided sufficient details on the random allocation method; however, two studies (22, 30) did not specify the randomization procedure, leading to a judgment of “unclear risk of bias” in this domain. Allocation concealment was explicitly described in two studies (21, 26), which were consequently rated as low risk of bias for this item. With respect to blinding, one study (21) reported blinding of outcome assessors, and another (26) blinded participants; both were judged to be at low risk of bias in the corresponding domains. Five RCTs (21, 23, 26, 27, 31) were considered to have low risk of bias for incomplete outcome data, as no missing outcome data were reported. One study (22) had registered its trial protocol, and no evidence of selective reporting was detected. No other sources of bias were identified. Overall, the predominant methodological limitation in this meta-analysis stems from insufficient reporting of randomization procedures (**Figure 2**).

### 3.4 Outcome measurements

For the quantitative synthesis, four studies (26–28, 31) reported partial non-normally distributed continuous variables as 25th -75th percentiles. Consequently, the corresponding outcome data from these studies were not amenable to meta-analysis, as the pooling of effect sizes typically requires means and standard deviations for continuous outcomes. Furthermore, although one study (27) initially enrolled 73 participants, only women with DOR who underwent IVF/ICSI were included in the present meta-analysis, comprising 16 in the acupuncture group and 17 in the control group.

#### 3.4.1 The primary outcomes

**Figure 3:**
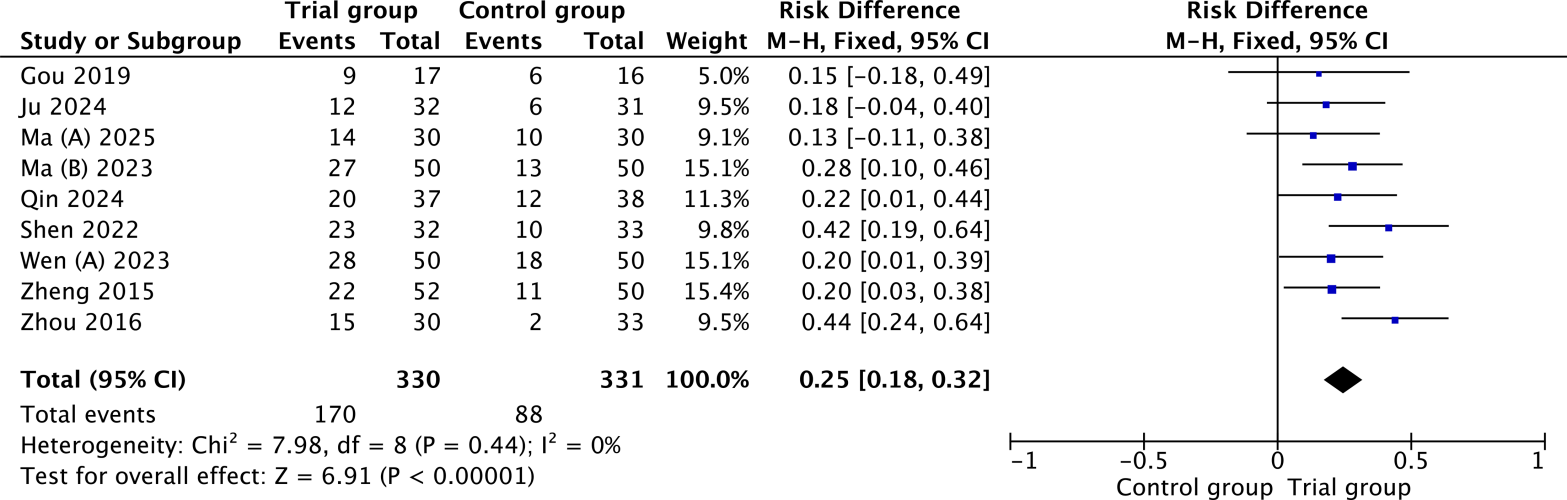
The primary outcomes.

Nine RCTs involving 661 women assessed the association between acupuncture and clinical pregnancy rate. Acupuncture was associated with a significant improvement in clinical pregnancy rate compared with the control group, with a pooled RD of 0.25, (95% CI: 0.18 to 0.32), I^2^ = 0%, *p* < 0.00001. Sensitivity analysis corroborated the robustness of this pooled estimate (**Figure 3A**, **Table 2**).

**Table 2:** Summary of findings.

Clinical outcomes	Studies (*n*)	Cases (*n*)	RD/MD 95% CI	*p*	I^2^ (%)	Model
Primary outcomes						
Clinical pregnancy rate	9	661	0.25 [0.18, 0.32]	< 0.00001	0	Fixed
Secondary outcomes						
Live birth rate	3	228	0.22 [0.05, 0.40]	0.01	57	Random
Number of oocytes retrieved	4	298	1.72 [1.03, 2.41]	< 0.00001	61	Random
Embryo implantation rate	3	283	0.30 [0.20, 0.41]	< 0.00001	46	Fixed
Number of optimal embryos	3	186	1.50 [1.42, 1.58]	< 0.00001	82	Random
Duration of Gn used	3	182	-0.05 [-0.70, 0.59]	0.87	28	Fixed
Total dose of Gn used	3	182	-12.22 [-253.01, 228.57]	0.92	0	Fixed
E_2_ on the day of hCG	2	99	318.48 [137.16, 499.80]	0.0006	23	Fixed
Resistance index	2	187	-0.06 [-0.13, 0.02]	0.16	74	Random
Pulsatility index	2	187	-0.14 [-0.39, 0.11]	0.28	83	Random
Systolic/Diastolic ratio	3	287	-0.40 [-1.00, 0.20]	0.19	79	Random
Subgroup analysis						
Clinical pregnancy rate (TEAS)	3	265	0.23 [0.12, 0.34]	< 0.0001	0	Fixed
Clinical pregnancy rate (Non-TEAS)	6	414	0.26 [0.17, 0.35]	< 0.00001	32	Fixed
Clinical pregnancy rate (≥ 10 acupoints)	5	363	0.27 [0.18, 0.37]	< 0.00001	37	Fixed
Clinical pregnancy rate (< 10 acupoints)	4	297	0.23 [0.12, 0.33]	< 0.0001	0	Fixed

#### 3.4.2 The secondary outcomes

**Figure 4:**
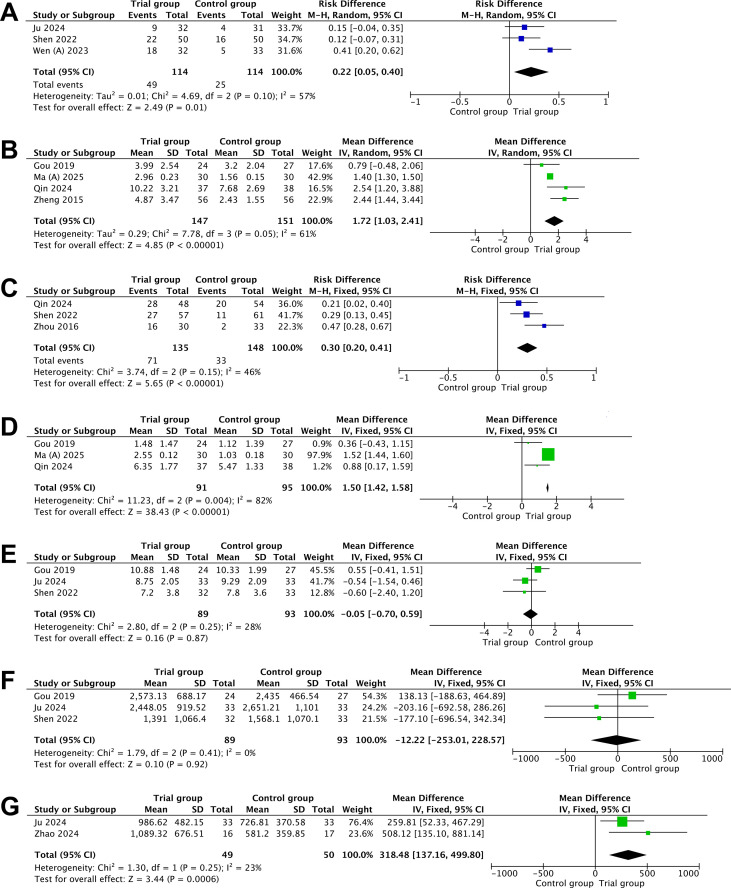
The secondary outcomes. **(A)** Live birth rate. **(B)** Number of oocytes retrieved. **(C)** Embryo implantation rate. **(D)** Number of optimal embryos. **(E)** Duration of gonadotropin used. **(F)** Total dose of gonadotropin used. **(G)** E_2_ on the day of hCG.

The live birth rate was investigated in 228 participants. The results found that acupuncture intervention was associated with a significant 22% rise in live birth rate (RD = 0.22, 95% CI: 0.05 to 0.40; I^2^ = 57%, *p* = 0.01) (**Figure 4A**). Furthermore, five studies assessed the value of acupuncture on the number of oocytes retrieved. Following exclusion of Wen’s study (29) in sensitivity analysis, I^2^ decreased from 89% to 61%. Among women with DOR undergoing IVF/ICSI, acupuncture was associated with a higher number of oocytes retrieved (MD = 1.72, 95% CI: 1.03 to 2.41; I^2^ = 61%, *p* < 0.00001) (**Figure 4B**). In addition, after exclusion of Zheng’s study (22) in sensitivity analysis, heterogeneity in embryo implantation rate decreased from 81% to 45%. The pooled analysis demonstrated a significant difference between groups, favoring acupuncture (RD = 0.30, 95% CI: 0.20 to 0.41; I^2^ = 45%, *p* < 0.00001) (**Figure 4C**).

Moreover, the number of optimal embryos was recorded in three trials. Pooled analysis implied that acupuncture may statistically increase the number of optimal embryos (MD = 1.50, 95% CI: 1.42 to 1.58; I^2^ = 82%, *p* < 0.00001) (**Figure 4D**). What’s more, four studies evaluated the effect of acupuncture on the duration of gonadotropin used and total dose of gonadotropin administered. Following sensitivity analysis with the exclusion of Zheng et al. (22), heterogeneity decreased from 80% to 0% for duration and from 88% to 28% for total dose. The results demonstrated no significant difference between the acupuncture and control groups for either duration of gonadotropin use (MD = -0.05, 95% CI: -0.70 to 0.59; I^2^ = 28%, *p* = 0.87) or total dose of gonadotropin used (MD = -12.22, 95% CI: -253.01 to 228.57; I^2^ = 0%, *p* = 0.92) (**Figures 4E, F**). In addition, E_2_ levels were considerably increased (MD = 318.48, 95% CI: 137.16 to 499.80; *p* = 0.0006) in the acupuncture group on the day of hCG trigger (**Figure 4G**).

**Figure 5:**
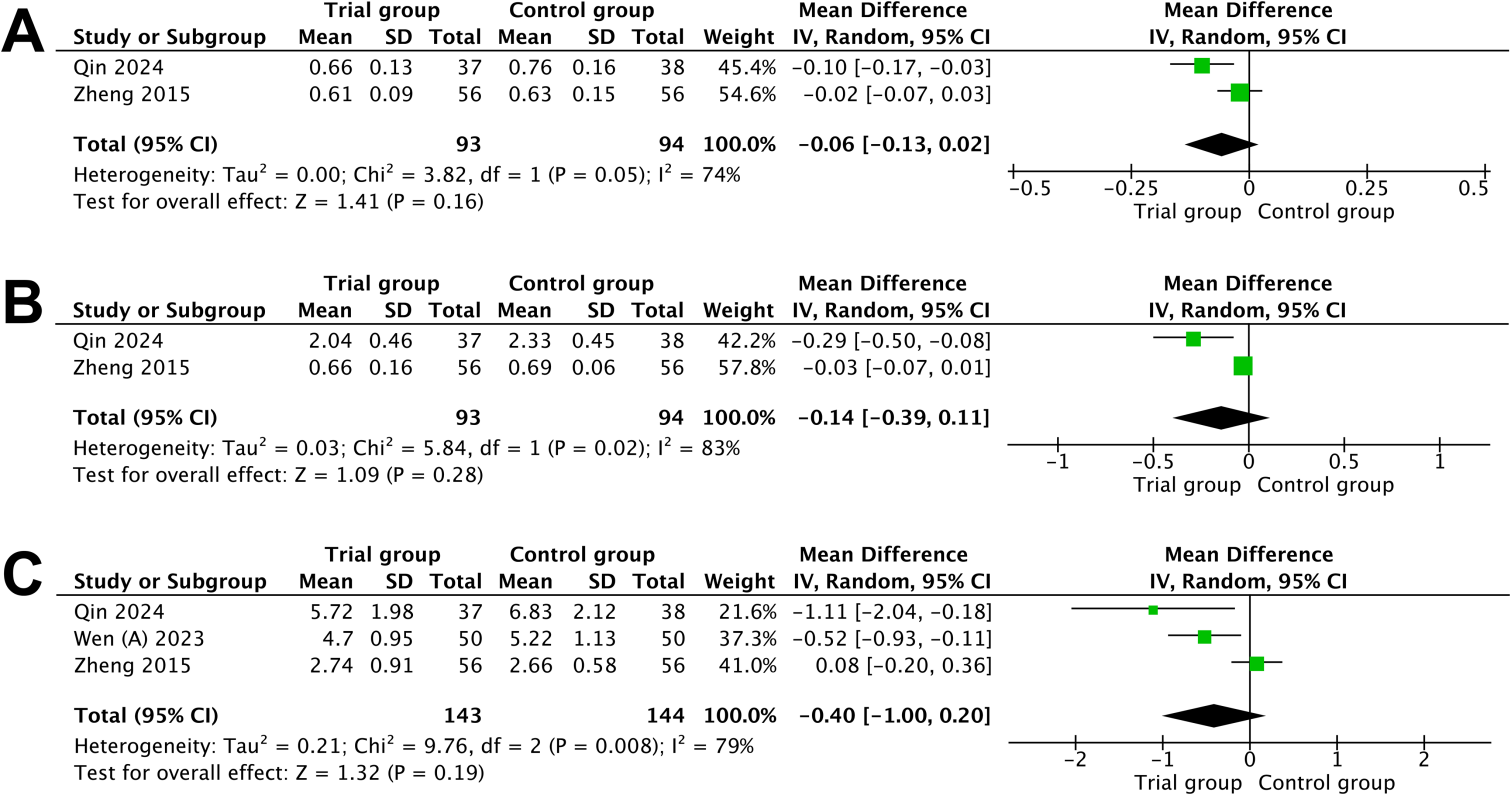
The secondary outcomes. **(A)** Resistance index. **(B)** Pulsatility index. **(C)** Systolic/Diastolic ratio of the ovarian arteries.

Regarding ultrasound indicators of the ovarian arteries, no significant effects were observed between the groups for resistance index (MD = -0.06, 95% CI: -0.13 to 0.02; I^2^ = 74%, *p* = 0.16) (**Figure 5A**), pulsatility index (MD = -0.14, 95% CI: -0.39 to 0.11; I^2^ = 83%, *p* = 0.28) (**Figure 5B**); systolic/diastolic ratio (MD = -0.40, 95% CI: -1.00 to 0.20; I^2^ = 79%, *p* = 0.19) (**Figure 5C**). **Table 2** summarizes the pooled results above.

#### 3.4.3 Adverse events

Beyond therapeutic efficacy, the safety of acupuncture intervention represents a critical consideration in clinical practice. However, adverse events (AEs) were reported in three of the 11 included RCTs. One study (26) documented no serious AEs during the intervention period. Mild bruising, mild pain, and mild allergic reactions were observed in the acupuncture groups in two trials (22, 29). The limited available data precluded a formal meta-analysis of AEs. Taken together, the available evidence suggests a favorable safety profile for acupuncture in women undergoing IVF/ICSI.

#### 3.4.4 Subgroup analysis

To comprehensively evaluate the clinical value of acupuncture for infertile women with DOR, this meta-analysis performed subgroup analyses based on the type of acupuncture intervention and the number of acupoints used per treatment session. Notably, although TEAS is a modern therapy derived from traditional acupuncture that does not involve needle insertion, it was categorized as an acupuncture-related intervention in this review, as it utilizes self-adhesive electrodes placed on the surface of acupoints, analogous to electroacupuncture and manual acupuncture in terms of acupoint stimulation (19).

**Figure 6:**
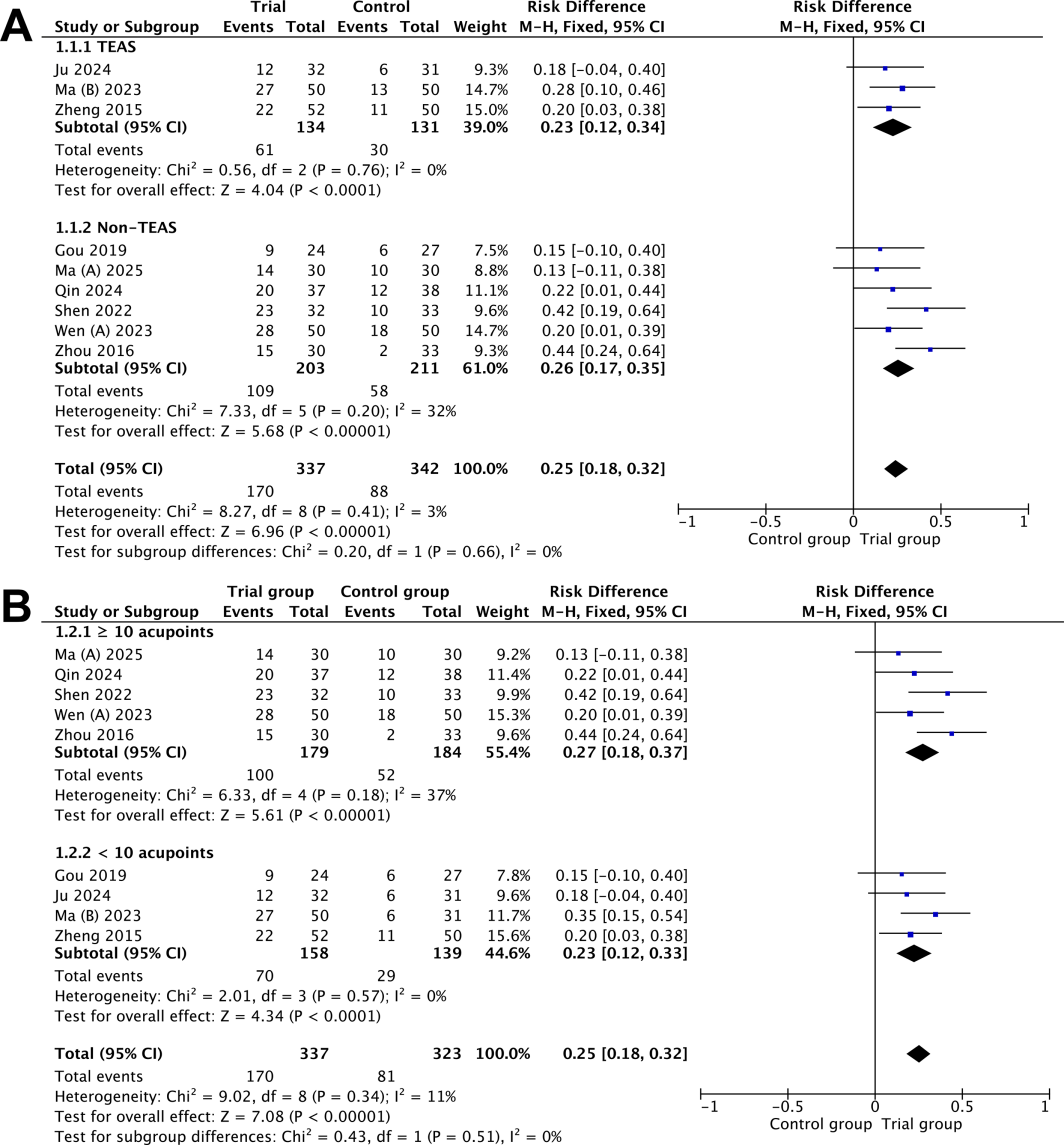
The results of subgroup analysis for clinical pregnancy rate. **(A)** TEAS *vs*. non-TEAS interventions. **(B)** ≥10 acupoints *vs*. < 10 acupoints.

To investigate whether different acupuncture modalities exert differential effects on clinical pregnancy rates, we first performed a subgroup analysis comparing TEAS with non-TEAS interventions. The pooled result demonstrated that both TEAS and non-TEAS interventions significantly increased clinical pregnancy rate, by 23% (RD = 0.23, 95% CI: 0.12 to 0.34; I^2^ = 0%, *p* < 0.0001) and 26% (RD = 0.26, 95% CI: 0.17 to 0.35; I^2^ = 32%, *p* < 0.00001), respectively (**Figure 6A**). Similarly, both high dose (≥10 acupoints) and low dose (< 10 acupoints) acupuncture regimens considerably increased clinical pregnancy rate by 27% (RD = 0.27, 95% CI: 0.18 to 0.37; I^2^ = 37%, *p* < 0.00001) and 23% (RD = 0.23, 95% CI: 0.12 to 0.33; I^2^ = 0%, *p* < 0.0001), respectively (**Figure 6A**). These findings are summarized in **Table 2**.

### 3.5 Certainty of the evidence

The overall certainty of evidence ranged from very low to moderate. In terms of inconsistency, comparisons for live birth rate, number of oocytes retrieved, number of optimal embryos, resistance index, pulsatility index, and systolic/diastolic ratio were downgraded by one level due to substantial heterogeneity (I^2^ > 50%). Except for clinical pregnancy rate, the remaining outcomes were further downgraded due to small sample sizes (*n* < 500). Additionally, other major considerations applied to all comparisons, as it was impossible to investigate potential publication bias using Begg’s and Egger’s tests because fewer than ten studies were available for each outcome (**Table 3**).

**Table 3:** Summary of GRADE evidence profiles.

Outcomes	Absolute effects (95% CI)	Relative effects (95% CI)	Number of cases	Quality of the evidence (GRADE)
Clinical pregnancy rate	25 more per 100 (from 15 more to 37 more)	RR 1.93 (1.58 to 2.38)	661	⨁⨁⨁◯^3^ Moderate
Live birth rate	20 more per 100 (from 13 more to 30 more)	RR 2.06 (1.09 to 3.93)	228	⨁◯◯◯^1,2,3^ Very low
Number of oocytes retrieved	MD 1.72 higher (1.03 higher to 2.41 higher)	–	298	⨁◯◯◯^1,2,3^ Very low
Embryo implantation rate	21 more per 100 (from 13 more to 31 more)	RR 2.37 (1.69 to 3.33)	283	⨁⨁◯◯^2,3^ Low
Number of optimal embryos	MD 1.50 higher (1.42 higher to 1.58 higher)	–	186	⨁◯◯◯^1,2,3^ Very low
Duration of Gn used	MD 0.05 lower (0.70 lower to 0.59 higher)	–	182	⨁⨁◯◯^2,3^ Low
Total dose of Gn used	MD 12.22 lower (253.01 lower to 228.57 higher)	–	182	⨁⨁◯◯^2,3^ Low
E_2_ on the day of hCG	MD 318.48 higher (137.16 higher to 499.80 higher)	–	99	⨁⨁◯◯^2,3^ Low
Resistance index	MD 0.06 lower (0.13 lower to 0.02 higher)	–	187	⨁◯◯◯^1,2,3^ Very low
Pulsatility index	MD 0.14 lower (0.39 lower to 0.11 higher)	–	187	⨁◯◯◯^1,2,3^ Very low
Systolic/Diastolic ratio	MD 0.40 lower (1.00 lower to 0.20 higher)	–	287	⨁◯◯◯^1,2,3^ Very low

^1^Downgraded one level for serious inconsistency: I^2^ > 50%.

^2^Downgraded one level for serious imprecision: small participants (*n*<500).

^3^Downgraded one level for other considerations: suspected publication bias.

## 4 Discussion

Despite the availability of various novel ovarian stimulation protocols, women with DOR often experience suboptimal outcomes following IVF/ICSI. Therefore, novel strategies to enhance IVF/ICSI outcomes in women with DOR are actively sought. Acupuncture, a non-pharmacological intervention, has emerged as a promising candidate, with studies elucidating its diverse biological mechanisms in reproduction (19). A recent study using a rat model of DOR demonstrated a significant association between acupuncture therapy and increased numbers of growing follicles, elevated E_2_ levels, and upregulated IL-4 and IL-10 expression, alongside reduced atretic follicle counts and decreased IFN-γ and TNF-α levels (33). These findings indicate that the beneficial effects of acupuncture may be attributed to the promotion of M2 macrophage polarization and the inhibition of inflammatory responses, ultimately leading to improved ovarian function (33). Furthermore, evidence from whole-transcriptome sequencing indicates that acupuncture exerts beneficial effects on ovarian function and early embryo development, potentially via the regulation of specific microRNAs such as miR-154-5p, miR-295-3p, miR-300-5p, miR-294-3p, miR-376c-5p, and miR-291a-3p in ovarian tissue or early-stage embryo tissues (34, 35). In addition, a recent study based on bisulfite sequencing illustrated that acupuncture can effectively increase the number of retrieved oocytes, and the potential mechanism was related to alterations in oocyte DNA methylation by targeting the calcium signaling pathways, Wnt, and, GnRH, along with Igf1r and Cdk5rap2 (36). Interestingly, acupuncture was found to modulate the activity of the bilateral superior frontal gyrus of women with DOR, normalizing its activity levels, and enhancing its functional connectivity with the lingual gyrus and bilateral calcarine sulcus as measured by magnetic resonance imaging (37).

### 4.1 Main results

To date, no comprehensive meta-analysis has been conducted to support the clinical application of acupuncture in women with DOR undergoing IVF/ICSI. Consequently, the clinical value of acupuncture in this population has not been fully elucidated. This meta-analysis involving 885 participants demonstrated that acupuncture is associated with improved reproductive outcomes, including significant increases in clinical pregnancy rate, live birth rate, number of oocytes retrieved, embryo implantation rate, number of optimal embryos, and E_2_ levels on the day of hCG. Sensitivity analyses corroborated the robustness of these results, implying that no single trial disproportionately influenced the pooled estimates. Clinically, managing infertile women with DOR undergoing IVF/ICSI poses a significant challenge, as this population often experiences low clinical pregnancy and live birth rates (38). The present meta-analysis demonstrates that acupuncture remarkably improves these outcomes, increasing clinical pregnancy rate by 25% and live birth rate by 22%, thereby providing clinicians with an additional therapeutic option for this unique population. A recent meta-analysis investigated adjuvant treatments and protocols for women with DOR undergoing ART, including dual stimulation, dehydroepiandrosterone, low-dose gonadotropin, and luteal phase stimulation. The authors reported that only 3 out of 13 protocols enhanced the number of oocytes retrieved (39). Therefore, identifying effective strategies to increase the number of oocytes retrieved remains a critical clinical challenge. This meta-analysis demonstrated that acupuncture offers a promising approach to address this issue (*p* < 0.00001). Interestingly, subgroup analyses revealed that both TEAS and non-TEAS interventions yielded comparable improvements in clinical pregnancy rate (23% *vs*. 26%). Similarly, regimens involving either ≥ 10 or < 10 acupoints per session were correlated with similar increases in clinical pregnancy rate (27% *vs*. 23%). Hence, subgroup analysis revealed that the effect of acupuncture on clinical pregnancy rate was consistent across different acupuncture modalities and varying numbers of acupoints. Furthermore, owing to limited reported data on AEs, a quantitative meta-analysis could not be performed. Nevertheless, three studies reported that AEs in the acupuncture groups were mild and transient, including bruising, pain, and allergic reactions. Importantly, no cases of ovarian hyperstimulation syndrome or other serious AEs were observed.

It is also interesting and innovative to compare the magnitude of the effect between acupuncture and other adjunctive therapies. A recent RCT divided women with DOR undergoing IVF into a control group receiving acupuncture and a trial group receiving acupuncture combined with TCM. The results demonstrated that acupuncture combined with TCM significantly increased the number of oocytes retrieved and the number of MII oocytes compared to the control group. However, no improvements were observed in the duration of Gn used, total dose of Gn used, embryo implantation rate, live birth rate, or clinical pregnancy rate. The previous study indicated that adding TCM to acupuncture could improve oocyte yield and maturation, but failed to enhance pregnancy related outcomes (40). Moreover, this meta-analysis indicated that acupuncture demonstrated no significant benefits on the duration and total dose of Gn stimulation, as well as resistance index, pulsatility index, and systolic/diastolic ratio of the ovarian arteries. The negative findings may be explained by the following reasons: First, primordial follicle pool depletion and impaired granulosa cell function in DOR are irreversible, and gonadotropin dosage and treatment duration are mainly determined by inherent ovarian reserve baseline. Second, acupuncture induced neurohumoral modulation is insufficient to reverse intrinsic ovarian responsiveness or alter standardized ovarian hyperstimulation protocols. Third, ovarian arterial hemodynamic parameters are dominated by ovarian interstitial fibrosis and vascular remodeling with low lability, which cannot be markedly modified by short term acupuncture intervention. Lastly, the limited number of eligible studies and clinical heterogeneity further restricted the statistical power to identify minor intergroup differences.

### 4.2 Limitations of this research

Although this is the first meta-analysis to investigate the clinical application of acupuncture in women with DOR undergoing IVF/ICSI, several limitations warrant consideration. First, despite acupuncture for women with DOR having been documented in several reports from different regions, including the United States (41) and Australia (10), only 11 RCTs conducted in China met our stringent inclusion criteria, potentially limiting the generalizability of these estimates to non-Chinese populations. Second, four studies reported skewed continuous variables using medians and 25th-75th percentiles. Although we attempted to convert these data into means and standard deviations, only one dataset followed a normal distribution, whereas the remaining data were still skewed. Pooling converted skewed data would increase the sample size but would also yield unreliable summary results. Therefore, to ensure methodological quality, these data were not incorporated into the meta-analysis. Third, cycle cancellation rate represents another critical outcome in IVF/ICSI treatment. However, none of the included RCTs reported this outcome; thus, the effect of acupuncture on cycle cancellation rate could not be estimated in this meta-analysis, which may represent an inherent limitation of the present study. Fourth, most of the included studies provided insufficient methodological descriptions. For instance, two studies merely described their allocation as “random” without further detail, nine did not report blinding procedures, and only one study had registered its trial protocol. These methodological limitations may introduce potential bias and thus should be considered when interpreting the findings of this meta-analysis. Fifth, the control groups involved heterogeneous interventions, including waitlist control, placebo TEAS, lifestyle counseling, and TCM. Besides, differences in concomitant ovarian stimulation protocols likely played a significant role. The included studies varied in whether minimal stimulation protocol, antagonist protocol, or conventional hormone treatment was used. Similarly, variation in acupuncture treatment parameters may have contributed to heterogeneity, which may downgrade the certainty of these findings. For example, some studies administered acupuncture for two weeks, while others delivered treatment across one to three menstrual cycles. Pooling data under such diverse clinical conditions inevitably introduces clinical heterogeneity and may potentially overestimate the therapeutic effects of acupuncture. Furthermore, most outcomes were reported by only two or three studies, which precluded further subgroup analysis to explore sources of heterogeneity owing to the limited number of eligible studies. Nevertheless, this meta-analysis provides a novel perspective on the role of acupuncture in improving clinical outcomes among women with DOR undergoing IVF/ICSI. Future trials may build on the present findings to further validate the efficacy of acupuncture in this population.

### 4.3 Implications for future research

First, a well-designed trial protocol is fundamental to generating robust evidence. However, only one of the included trial stated protocol registration in an established trial registry, suggesting that the majority may not have conducted comprehensive outcome assessments. Future RCTs should prioritize standardized outcome reporting in accordance with guidelines such as CONSORT (42). Second, although factors such as age, BMI, and duration of infertility may influence the effect of acupuncture on IVF/ICSI outcomes, none of the included studies compared these variables. Therefore, whether the effectiveness of acupuncture differs according to these baseline characteristics warrants further investigation. Third, given that only nine of the 11 included RCTs reported clinical pregnancy rates, a formal assessment of publication bias using Begg’s test could not be performed, as the *Cochrane Handbook* recommends a minimum of 10 studies for this analysis. Therefore, further large-scale, multicenter trials are warranted to validate these findings and enhance the current evidence. Promisingly, numerous clinical trial protocols investigating the role of acupuncture for women with DOR undergoing IVF/ICSI have been published recently. The present meta-analysis may be updated in the future by incorporating the results of these ongoing trials once they become available.

## 5 Conclusion

The accumulated evidence demonstrates that acupuncture may favorably influence clinical pregnancy rate, live birth rate, number of oocytes retrieved, embryo implantation rate, number of optimal embryos, and serum E_2_ levels on the day of hCG. However, several findings are limited by small sample sizes, substantial heterogeneity, wide confidence intervals, as well as very low certainty of evidence and should be interpreted cautiously. Therefore, additional large-scale trials conducted in diverse geographic regions and adhering to guidelines such as CONSORT are warranted to further corroborate the clinical value of acupuncture in this population.

## Data Availability

The original contributions presented in the study are included in the article/**Supplementary Material**. Further inquiries can be directed to the corresponding author.

## References

[1] Boivin J Bunting L Collins JA Nygren KG . International estimates of infertility prevalence and treatment-seeking: potential need and demand for infertility medical care. Hum Reprod. (2007) 22:1506–12. doi:10.1093/humrep/dem046 17376819

[2] Liang Y Huang J Zhao Q Mo H Su Z Feng S . Global, regional, and national prevalence and trends of infertility among individuals of reproductive age (15-49 years) from 1990 to 2021, with projections to 2040. Hum Reprod. (2025) 40:529–44. doi:10.1093/humrep/deae292 39752330

[3] Bell SO Moreau C Sarnak D Kibira SPS Anglewicz P Gichangi P . Measuring non-events: infertility estimation using cross-sectional, population-based data from four countries in sub-Saharan Africa. Hum Reprod. (2024) 39:2848–60. doi:10.1093/humrep/deae218 39348340 PMC11629970

[4] Zhou Z Zheng D Wu H Li R Xu S Kang Y . Epidemiology of infertility in China: a population-based study. BJOG. (2018) 125:432–41. doi:10.1111/1471-0528.14966 29030908

[5] Morshed-Behbahani B Lamyian M Joulaei H Rashidi BH Montazeri A . Infertility policy analysis: a comparative study of selected lower middle- middle- and high-income countries. Global Health. (2020) 16:104. doi:10.1186/s12992-020-00617-9 33097089 PMC7583186

[6] Yoshida T Kikuchi I Sako Y Kitano T Hirata T . Japanese health insurance coverage of fertility treatment in 2022: does coverage change patient perspectives? Cureus. (2024) 16:e74459. doi:10.7759/cureus.74459 39726454 PMC11669821

[7] Carson SA Kallen AN . Diagnosis and management of infertility: A review. JAMA. (2021) 326:65–76. doi:10.1001/jama.2021.4788 34228062 PMC9302705

[8] Maunder A Arentz S Armour M Costello MF Ee C . Health needs, treatment decisions and experience of traditional complementary and integrative medicine use by women with diminished ovarian reserve: A cross-sectional survey. Aust N Z J Obstet Gynaecol. (2024) 64:390–8. doi:10.1111/ajo.13805 38514899

[9] Devine K Mumford SL Wu M DeCherney AH Hill MJ Propst A . Diminished ovarian reserve in the United States assisted reproductive technology population: diagnostic trends among 181,536 cycles from the Society for Assisted Reproductive Technology Clinic Outcomes Reporting System. Fertil Steril. (2015) 104(3):612–19.e3. doi:10.1016/j.fertnstert.2015.05.017 26049057 PMC4560955

[10] Busnelli A Somigliana E Cirillo F Levi-Setti PE . Is diminished ovarian reserve a risk factor for miscarriage? Results of a systematic review and meta-analysis. Hum Reprod Update. (2021) 27:973–88. doi:10.1093/humupd/dmab018 34254138

[11] Kawwass JF Hipp HS Session DR Kissin DM Jamieson DJ National ART Surveillance System Group . Severity of diminished ovarian reserve and chance of success with assisted reproductive technology. J Reprod Med. (2017) 62:153–60. PMC11057004 30230307

[12] Lin G Zhong X Li S Liu X Xu L . The clinical value of progestin-primed ovarian stimulation protocol for women with diminished ovarian reserve undergoing IVF/ICSI: a systematic review and meta-analysis. Front Endocrinol (Lausanne). (2023) 14:1232935. doi:10.3389/fendo.2023.1232935 37670890 PMC10476097

[13] Lin G Li X Jin YS Xu L . Clinical evidence of coenzyme Q10 pretreatment for women with diminished ovarian reserve undergoing IVF/ICSI: a systematic review and meta-analysis. Ann Med. (2024) 56:2389469. doi:10.1080/07853890.2024.2389469 39129455 PMC11321116

[14] Lin G Zhong X Li S Xu L . Clinical evidence of growth hormone for infertile women with diminished ovarian reserve undergoing IVF: a systematic review and meta-analysis. Front Endocrinol (Lausanne). (2023) 14:1215755. doi:10.3389/fendo.2023.1215755 38027219 PMC10663944

[15] Lin G Liu X Cong C Chen S Xu L . Clinical efficacy of acupuncture for diminished ovarian reserve: a systematic review and meta-analysis of randomized controlled trials. Front Endocrinol (Lausanne). (2023) 14:1136121. doi:10.3389/fendo.2023.1136121 37600702 PMC10433735

[16] Zhang R Lin G Wang W . Clinical evidence of acupuncture for luteinized unruptured follicle syndrome: a systematic review and meta-analysis of randomized controlled trials. Front Endocrinol (Lausanne). (2025) 16:1640820. doi:10.3389/fendo.2025.1640820 40951412 PMC12425743

[17] Liu Y Wei M Deng Y Fan Y Zheng Y Ni Z . Advances in traditional chinese medicine for managing diminished ovarian reserve: mechanisms and clinical insights. Drug Des Devel Ther. (2025) 19:5597–614. doi:10.2147/DDDT.S505689 40620463 PMC12228491

[18] Huang S Zhang D Shi X Zhang Y Wang X She Y . Acupuncture and related therapies for anxiety and depression in patients with premature ovarian insufficiency and diminished ovarian reserve: a systematic review and meta-analysis. Front Psychiatry. (2024) 15:1495418. doi:10.3389/fpsyt.2024.1495418 39687777 PMC11647530

[19] Qu F Li R Sun W Lin G Zhang R Yang J . Use of electroacupuncture and transcutaneous electrical acupoint stimulation in reproductive medicine: a group consensus. J Zhejiang Univ-Sci B. (2017) 18:186–93. doi:10.1631/jzus.B1600437 28271655 PMC5369245

[20] Ma L Zhang M Tang H . Discussion on the effect of warm acupuncture on IVF-ET in infertile patients with diminished ovarian reserve based on the theory of "Mingmen, Yuanqi, Sanjiao. Hunan J Tradit Chin Med. (2025) 41:18–22. doi:10.16808/j.cnki.issn1003-7705.2025.03.005

[21] Gou W . A randomized controlled trial on the effect of acupuncture in IVF-ET for infertile patients with diminished ovarian reserve. In: Lanzhou university. Lanzhou (2019).

[22] Zheng Y Feng X Mi H Yao Y Zhao Y Li J . Effects of transcutaneous electrical acupoint stimulation on ovarian reserve of patients with diminished ovarian reserve in *in vitro* fertilization and embryo transfer cycles. J Obstet Gynaecol Res. (2015) 41:1905–11. doi:10.1111/jog.12810 26455718

[23] Qin C Jiang J Zhong Y . Effect of electroacupuncture cycle therapy on endometrial receptivity and *in vitro* fertilization-embryo transfer in infertile patients with diminished ovarian reserve. J Mod Integr Med. (2024) 33:785–90.

[24] Page MJ McKenzie JE Bossuyt PM Boutron I Hoffmann TC Mulrow CD . The PRISMA 2020 statement: an updated guideline for reporting systematic reviews. BMJ. (2021) 372:n71. doi:10.1136/bmj.n71 33782057 PMC8005924

[25] Expert Group of Consensus on Clinical Diagnosis & Management of Diminished Ovarian Reserve Reproductive Endocrinology & Fertility Preservation Section of Chinese Society on Fertility Preservation Chinese Preventive Medicine Association . Consensus on clinical diagnosis and management of diminished ovarian reserve. J Reprod Med. (2022) 31:425–34. doi:10.1055/s-0033-1356472 3785596

[26] Ju W . Study on the mechanism of transcutaneous acupoint stimulation regulating granulosa cell apoptosis in patients with diminished ovarian reserve of kidney deficiency type. In: Shandong university of traditional chinese medicine. Jinan (2024). doi:10.27282/d.cnki.gsdzu.2024.000066

[27] Zhao Y . Clinical study on acupuncture improving diminished ovarian reserve and exploration of its mechanism based on miR212-3p/mTORC1 signaling pathway. In: Chengdu university of traditional chinese medicine. Chengdu (2024). doi:10.26988/d.cnki.gcdzu.2024.000038

[28] Wen C Zhang X Wang Z Li L Li J Guan Y . Effect of acupuncture on pregnancy outcomes in hormone replacement therapy-frozen-thawed embryo transfer cycles for infertility patients with diminished ovarian reserve. J Tianjin Univ Tradit Chin Med. (2023) 42:692–6. doi:10.11656/j.issn.1673-9043.2023.06.03

[29] Wen C Zhang X Wan L Li L Li J Guan Y . Clinical study on acupuncture for infertility with diminished ovarian reserve. J New Chin Med. (2023) 55:127–31. doi:10.13457/j.cnki.jncm.2023.02.031

[30] Ma L Zheng X Yang L Li L Mei J Li J . Observation on clinical efficacy of transcutaneous acupoint electrical stimulation combined with Chinese herbal medicine for tonifying kidney and activating blood circulation in patients with diminished ovarian reserve undergoing assisted reproductive technology. Mod Diagn Treat. (2023) 34:1–4,7.

[31] Shen J Gao Y Lu G Chen L Cheng J Xia Y . Effect of electroacupuncture on endometrial receptivity and IVF-ET pregnancy outcome in patients with diminished ovarian reserve. Chin Acupunct Moxibust. (2022) 42:879–83. doi:10.13703/j.0255-2930.20210901-k0002 35938330

[32] Zhou L Xia Y Ma X Tang L Lu J Tang Q . Effect of sequential acupuncture therapy on IVF-ET in patients with diminished ovarian reserve. Chin Acupunct Moxibust. (2016) 36:25–8. doi:10.13703/j.0255-2930.2016.01.007 26946729

[33] Luo J Qin Y Zhu Y Yin Y Shen M . Electroacupuncture improves ovarian function in rats with tripterygium glycoside-induced diminished ovarian reserve by promoting the polarization of M2 macrophages and inhibiting inflammatory responses. Mediators Inflammation. (2025) 2025:1694470. doi:10.1155/mi/1694470 40201729 PMC11976048

[34] Hao J Zhao Y Liu X Xu H Liu L Wang H . Whole-transcriptome sequencing reveals the effects of acupuncture on early embryos post-IVF-ET in poor ovarian response. J Ovarian Res. (2025) 18:91. doi:10.1186/s13048-025-01682-7 40307905 PMC12044900

[35] Liu Y Yang W Yuan R Li Z Wang T Yang B . Exploring the mechanism of acupuncture in improving ovarian function in rats with poor ovarian response using high-throughput sequencing. Comb Chem High Throughput Screen. (2025) 28:1443–57. doi:10.2174/0113862073365843241223093834 39838669

[36] Wang M Li Z Xiong Y Yuan R Zhu X Chen X . Acupuncture increased the number of retrieved oocytes in a mouse model of POR: the involvement of DNA methylation in the oocytes. Comb Chem High Throughput Screen. (2025) 28:132–45. doi:10.2174/0113862073264460231113052942 39957304

[37] Tianyu B Jiaen Y Liang Y Jinling LI Jianxian L Zongchang LI . Effect of acupuncture on brain activity in patients with decreasing ovarian reserve: a resting-state functional magnetic resonance imaging study. J Tradit Chin Med. (2025) 45:450–7. doi:10.19852/j.cnki.jtcm.2025.02.011 40151132 PMC11955760

[38] Zhu S Jiang W Liao X Sun Y Chen X Zheng B . Effect of diminished ovarian reserve on the outcome of fresh embryo transfer in IVF/ICSI cycles among young women: A retrospective cohort study. BMC Womens Health. (2024) 24:230. doi:10.1186/s12905-024-03039-6 38594688 PMC11003098

[39] Conforti A Carbone L Di Girolamo R Iorio GG Guida M Campitiello MR . Therapeutic management in women with a diminished ovarian reserve: a systematic review and meta-analysis of randomized controlled trials. Fertil Steril. (2025) 123:457–76. doi:10.1016/j.fertnstert.2024.09.038 39332623

[40] He X Zhou Y Li L Jiang L Hu Y . Clinical observation of yulinzhu decoction combined with yin-yang acupuncture in treatment of pregnancy outcome of patients with decreased ovarian reserve function in the fourth group of poseidon treated with IVF-ET. Parmacol Clin Chin Mater Med. (2024) 40:89–93.

[41] Hullender Rubin L Marx BL . Diminished ovarian reserve, clomid, and traditional chinese medicine: A case study. Med Acupunct. (2012) 24:273–80. doi:10.1089/acu.2012.0912 24761166 PMC3579201

[42] Hopewell S Chan AW Collins GS Hróbjartsson A Moher D Schulz KF . CONSORT 2025 statement: updated guideline for reporting randomised trials. BMJ. (2025) 389:e081123. doi:10.1136/bmj-2024-081123 40228833 PMC11995449

