# Supplementary material for: Clinical evidence of acupuncture for infertile women with diminished ovarian reserve undergoing IVF/ICSI: a systematic review and meta-analysis
Source: Front Endocrinol (Lausanne). 2026 Jun 10;17:1840157. doi: 10.3389/fendo.2026.1840157 (PMC13290564; doi:10.3389/fendo.2026.1840157)
Supplement: Supplementary file 1 [file Table1.docx]

TABLE 1 Study main characteristics.

| Study | Year | Sample size (***n***) | | Regimen of treatment | | No. of acupoints | Ovulation induction protocol | Duration of treatment |
| --- | --- | --- | --- | --- | --- | --- | --- | --- |
|  |  | Control | Trial | Trial | Control |  |  |  |
| Ma (A) (20) | 2025 | 30 | 30 | Manual acupuncture | Waitlist | 21 | Minimal stimulation protocol | 1.5 months |
| Ju (26) | 2024 | 33 | 33 | TEAS | Placebo TEAS | 6 | Antagonist protocol | One menstrual cycle |
| Zhao (27) | 2024 | 36 | 37 | Manual acupuncture | Lifestyle counseling | 14 | Minimal stimulation protocol | 8 to12 weeks |
| Qin (23) | 2024 | 38 | 37 | Manual acupuncture | Waitlist | 16 | Minimal stimulation protocol | Three menstrual cycles |
| Wen (A) (28) | 2023 | 50 | 50 | Manual acupuncture | Waitlist | 14 | HRT | Two weeks |
| Wen (B) (29) | 2023 | 60 | 60 | Manual acupuncture | Waitlist | 14 | HRT | 12 weeks |
| Ma (B) (30) | 2023 | 50 | 50 | TCM + Manual acupuncture | TCM | 5 | Antagonist protocol | Three menstrual cycles |
| Shen (31) | 2022 | 33 | 32 | Electroacupuncture | Waitlist | 15 | HRT | Three menstrual cycles |
| Gou (21) | 2019 | 27 | 24 | Manual acupuncture | Waitlist | 9 | Minimal stimulation protocol | Two menstrual cycles |
| Zhou (32) | 2016 | 33 | 30 | Manual acupuncture | Waitlist | 13 | Minimal stimulation protocol | Two menstrual cycles |
| Zheng (22) | 2015 | 56 | 56 | TEAS | Placebo TEAS | 8 | Not reported | Three menstrual cycles |

Abbreviations: TCM, Traditional Chinese medicine; TEAS, Transcutaneous electrical acupoint stimulation; HRT, Hormone replacement therapy.
